# P-947. Evaluation of Priorities and Inclusion in Coaching Opportunities for Infectious Disease Advanced Practice Providers (APPs)

**DOI:** 10.1093/ofid/ofae631.1137

**Published:** 2025-01-29

**Authors:** Alison M Beieler, Leah H Yoke, Andrea J Zimmer, Luke Strnad, David J Riedel

**Affiliations:** Harborview Medical Center, Seattle, Washington; University of Washington; Fred Hutch Cancer Research Center, Seattle, WA; University of Nebraska Medical Center, Omaha, NE; Oregon Health and Science University, Portland, Oregon; Institute of Human Virology, University of Maryland School of Medicine, Baltimore, MD

## Abstract

**Background:**

In line with the Infectious Diseases Society of America (IDSA) mission statement, the IDSA Medical Education of Community Practice launched in 2019 establishing several sub-groups, including a mentoring workgroup. This group successfully started a Clinician Educator Coaching Program for Infectious Disease (ID) in which junior faculty are paired with experienced faculty to meet around IDWeek. Advanced practice providers (APPs) are a growing workforce within ID but have limited access to formal mentoring programs despite expressed desire for mentorship. In response, the IDSA APP interest group developed a survey to identify APP priorities and then developed a pilot coaching program to address some of the mentoring needs identified.

**Figure 1.**
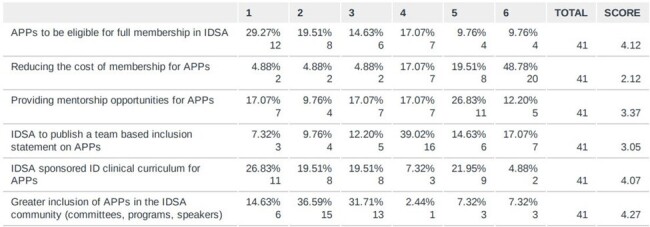
Anonymous survey of six APP priorities ranked by score (n = 41)

**Methods:**

An anonymous survey was distributed via email and the IDSA IDea exchange in 2022 to evaluate APP priorities on topics including education, mentorship, inclusion, and IDSA membership. Following that survey, an APP coach/advisee pilot program was created and advertised via email and the IDSA IDea exchange from 8/16/23 – 9/1/23. APP coaches were defined as those with > 6 years ID experience while APP advisees were those in practice < 3 years. Coaches and advisees were paired and encouraged to meet virtually over several sessions.

**Table 1.**
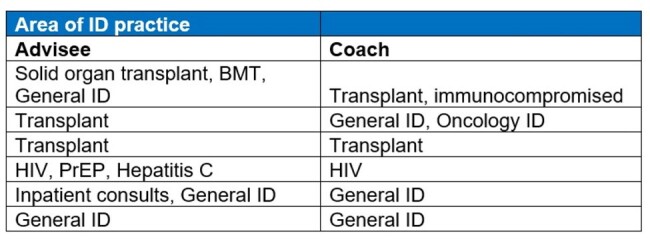
Matches for APP Advisee/Coach by area of ID practice. (n=6) BMT: bone marrow transplant, HIV: human immunodeficiency virus, PrEP: Pre-exposure prophylaxis

**Results:**

41 survey responses were returned from APPs in ID with six ranked priorities using calculated scoring (Figure 1). Overall, “mentorship” was the 3rd most common item scored as a number one priority, and 44% of the respondents ranked it as one of their top three priorities. Six APP coach/advisee pairs were matched, based on specialty of ID practice (Table 1). An advisee survey identified individual needs assessment pre-meeting (Table 2). Feedback from participants was limited mostly to coaches by response rates post-meeting (5 coaches, 1 advisee), but suggested the coaching program was beneficial overall (Table 3).

**Table 2.**
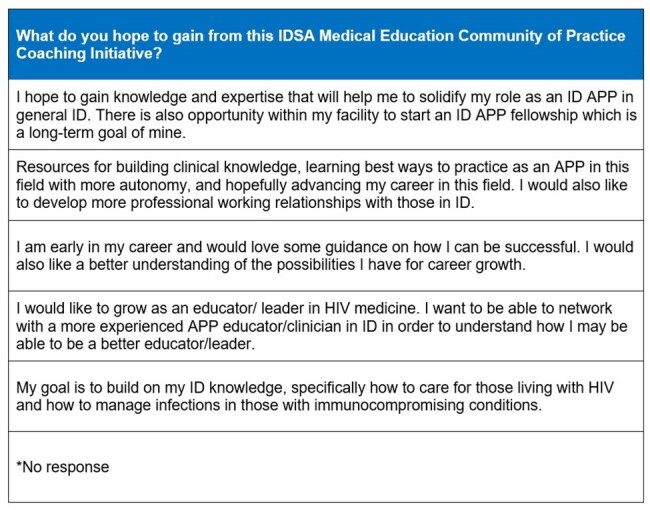
APP Advisee Pre-Meeting Needs Assessment (n = 6)

**Conclusion:**

APP priorities included IDSA membership, education, inclusion, and mentorship. While formal APP mentorship has not yet been established in ID, a pilot APP ID coaching program launched successfully with six pairs. Additional APP coaching or mentoring opportunities alongside other educational curriculum could be offered within IDSA to meet needs and optimally support APPs in ID.

**Table 3.**
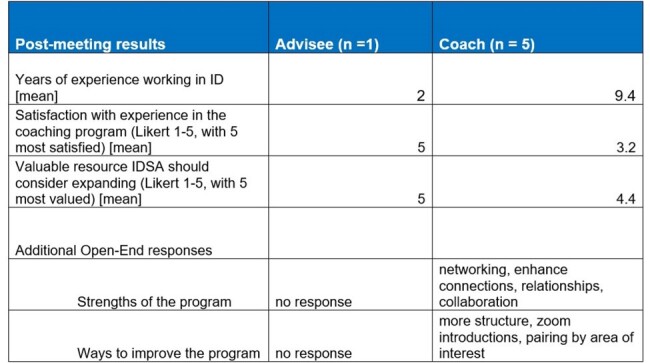
APP Coaching Pilot Post-Meeting Survey Results

**Disclosures:**

**All Authors**: No reported disclosures

